# Insights into the Role of Plasmatic and Exosomal microRNAs in Oxidative Stress-Related Metabolic Diseases

**DOI:** 10.3390/antiox12061290

**Published:** 2023-06-16

**Authors:** Ayauly Duisenbek, Gabriela C. Lopez-Armas, Miguel Pérez, María D. Avilés Pérez, José Miguel Aguilar Benitez, Víctor Roger Pereira Pérez, Juan Gorts Ortega, Arailym Yessenbekova, Nurzhanyat Ablaikhanova, Germaine Escames, Darío Acuña-Castroviejo, Iryna Rusanova

**Affiliations:** 1 Department of Biophysics, Biomedicine and Neuroscience, Al-Farabi Kazakh National University, Al-Farabi Av. 71, Almaty 050040, Kazakhstan; ayauly.duisenbek@kaznu.edu.kz (A.D.); nurzhanat75@mail.ru (N.A.); 2 Department of Biochemistry and Molecular Biology I, Faculty of Science, University of Granada, 18019 Granada, Spain; 3 Departamento de Investigación y Extensión, Centro de Enseñanza Técnica Industrial, C. Nueva Escocia 1885, Guadalajara 44638, Mexico; glopez@ceti.mx; 4 Hospital de Alta Resolución de Alcalá la Real, 23680 Jaén, Spain; miguel.perez.porras.sspa@juntadeandalucia.es (M.P.); josemiguel.aguilar.sspa@juntadeandalucia.es (J.M.A.B.); 5 Endocrinology and Nutrition Unit, Instituto de Investigación Biosanitaria de Granada (Ibs.GRANADA), University Hospital Clínico San Cecilio, 18016 Granada, Spain; mariolaviles@live.com; 6 Centro de Investigación Biomédica en Red Fragilidad y Envejecimiento Saludable (CIBERfes), Instituto de Investigación Biosanitaria de Granada (Ibs.GRANADA), San Cecilio University Hospital Clínico, 18016 Granada, Spain; gescames@ugr.es (G.E.); dacuna@ugr.es (D.A.-C.); 7 Instituto de Biotecnología, Centro de Investigación Biomédica, Parque Tecnológico de Ciencias de la Salud, Universidad de Granada, 18016 Granada, Spain; 8 Department of Physiology, Faculty of Medicine, University of Granada, 18016 Granada, Spain

**Keywords:** metabolic diseases, circulating microRNA, oxidative stress, exosomes, epigenetic, inflammation, endothelial dysfunction

## Abstract

A common denominator of metabolic diseases, including type 2 diabetes Mellitus, dyslipidemia, and atherosclerosis, are elevated oxidative stress and chronic inflammation. These complex, multi-factorial diseases are caused by the detrimental interaction between the individual genetic background and multiple environmental stimuli. The cells, including the endothelial ones, acquire a preactivated phenotype and metabolic memory, exhibiting increased oxidative stress, inflammatory gene expression, endothelial vascular activation, and prothrombotic events, leading to vascular complications. There are different pathways involved in the pathogenesis of metabolic diseases, and increased knowledge suggests a role of the activation of the NF-kB pathway and NLRP3 inflammasome as key mediators of metabolic inflammation. Epigenetic-wide associated studies provide new insight into the role of microRNAs in the phenomenon of metabolic memory and the development consequences of vessel damage. In this review, we will focus on the microRNAs related to the control of anti-oxidative enzymes, as well as microRNAs related to the control of mitochondrial functions and inflammation. The objective is the search for new therapeutic targets to improve the functioning of mitochondria and reduce oxidative stress and inflammation, despite the acquired metabolic memory.

## 1. Introduction

Metabolic syndrome is a complex condition that includes a set of vascular risk factors such as abdominal obesity, insulin resistance, hypertension, impaired glucose metabolism, and abnormal cholesterol and triglyceride levels [1]. Metabolic syndrome affects approximately 20–30% of adults worldwide and increases the risk of type 2 diabetes mellitus (T2DM), cardiovascular diseases, and nonalcoholic fatty liver disease. Although the precise reasons for metabolic syndrome are not entirely understood, it is widely believed to be caused by an aggregate of genetic factors and lifestyle choices such as poor diet, sedentary lifestyles, and certain medications such as corticosteroids and antipsychotic drugs [2]. One clear example of this interaction with the external environment is the ingestion of advanced glycation end products (AGEs) exogenous to the modern Western diet. The thermal processing of food and conservation and safety processes of food lead to the generation of diverse food-derived AGEs known as glycotoxins [3,4]. These exogenous AGEs contribute to the internal AGEs pool and will contribute to the development of metabolic memory [5]. 

Metabolic oxidative stress-related diseases include T2DM, cardiovascular complications related to atherosclerosis, and nonalcoholic fatty liver disease, which are associated with high mortality and morbidity. A common denominator of metabolic diseases is a high body mass index or/and prolonged exposure to external factors that modify the epigenetics of the organism. Epigenetics is heritable gene expression and function changes that affect the non-coding acid nucleic material without changes in the underlying DNA sequence [6]. The epigenetic modifications include DNA methylation, histone modifications, and changes in non-coding RNAs, which can alter gene transcription in response to environmental stimuli. 

Two long-time studies in humans (“The Diabetes Control and Complications Trial” (DCCT) (1983–1993) and a follow-up one, namely, «Epidemiology of Diabetes Interventions and Complications» (EDIC) (1994 to present)) demonstrated that intensive therapy at the early stage of diabetes mellitus 1 (DM1) disease prevents or significantly slows the appearance and progression of microvascular complications (retinopathy, neuropathy, and nephropathy), as well as macrovascular ones (stroke, atherosclerosis, and heart ischemic disease), compared to patients who received one conventional control therapy [7,8]. Follow-up of these patients for almost 10 years demonstrated a persistent beneficial effect of early glycemic control on the progression of macrovascular abnormalities (such as carotid artery intima-media thickness) and a significant reduction in the risk of fulminant myocardial infarction, cerebrovascular accident, or death due to cardiovascular disease. These findings suggest that early tight control could benefit both the micro- and macro vasculature despite the average HbA1c levels in the groups remaining almost equivalent. Metabolic memory is also produced in T2DM, as demonstrated in the United Kingdom Prospective Diabetes Study (UKPDS) study, which included 5102 patients recently diagnosed with T2DM and with a median follow-up of 10 years [9].

On the other hand, “metabolic programming” has been described during the early stages of an individual’s life history that will influence the development of metabolic complications in the future. On the other hand, the changes that occur in the early stage of metabolic diseases will be difficult to treat after being diagnosed. For example, prolonged endothelial cell exposure to chronic hyperglycemia leads to endothelial dysfunction caused by the appearance of metabolic memory before T2DM is diagnosed in the clinic, which, despite reasonable subsequent glycemic control in later years, may lead to T2DM complications [10]. Studying epigenetic changes in different metabolic pathways involved in metabolic memory and vascular complications’ appearance is becoming increasingly important. 

Epigenetic histone post-translational modifications were assessed in white blood cells from a subset of DCCT/EDIC participants, including 30 case subjects from the DCCT conventional treatment group. These case subjects had a high mean DCCT HbA1c and progression of retinopathy or nephropathy in their 10th year of follow-up, and 31 control subjects received intensive glycemic treatment, without EDIC complications, in their 10th year of follow-up. Monocytes from case patients presented statistically more significant numbers of promoter regions with H3 lysine-9 acetylation (H3K9Ac). Taking into account that histone hyperacetylation can promote chromatin relaxation and gene expression, these promoters had the active mark, increasing gene expression, and more than 15 genes were related to the nuclear factor-kappa light chain enhancer of the activated B cells’ (NF-κB) inflammatory pathway [11]. This was one of the first human discoveries that linked the glycemic history (HbA1c) to epigenetic level changes in the promoters of gene pathways related to disease progression. 

Several studies show that glucose-mediated changes in gene expression largely persist in people with diabetes, despite reversing hyperglycemia. Al-Dabet et al. demonstrated the role of p21 as a mediator and biomarker of hyperglycemic memory in diabetic kidney disease (DKD) [12]. Increased p21 expression was detected in various animal models, human samples, and in vitro models, remaining sustained despite glucose-lowering by SGLT2l or insulin. The proposed mechanism for regulating persistent tubular p21 expression was linked to the demethylation of its promoter by reduced DNA methyltransferase 1 (DNMT1) expression [12]. DNMT1 induces p21 promoter methylation and reduces p21 expression. The importance lies in the fact that p21 is closely associated with senescence and other senescence markers, such as induction of senescence-associated secretory phenotype (SASP) genes (IL-1b, IL-6) and of cell-cycle kinase inhibitors (p15, p16, p19) [13].

Evidence suggests that epigenetic mechanisms such as DNA methylation or post-translational histone modifications are mechanistically linked to hyperglycemic memory [14]. Epigenetic modifications include not only chromatin changes (such as DNA and histone methylation, histone acetylation, and N6-methyladenosine (m6A) modification) but also modifications to non-coding RNAs (ncRNAs) [15,16]. The regulatory ncRNAs are divided into two categories based on size: short-chain ncRNAs (including microRNAs [miRNAs], etc.) and long non-coding RNAs (lncRNAs).

This review will focus on the regulatory non-coding RNAs as ideal candidates for novel molecular markers and therapeutic strategies for preventing and treating metabolic memory in metabolic syndrome. 

First, we will review various metabolic pathways involved in the metabolic syndrome. A chronic low-grade inflammatory state is known to accompany obesity and is a common factor in metabolic diseases. Growing evidence suggests that chronic inflammation and high oxidative stress impact all organisms’ metabolic homeostasis, causing insulin resistance and metabolic reprogramming of different organs. At the pancreas level, these changes disturb the production of insulin by beta cells. At the level of insulin-dependent cells, insulin resistance occurs.

Moreover, in non-insulin-dependent cells, especially in the endothelium, these changes cause endothelial dysfunction and the appearance of cardiovascular complications. Accumulating evidence has also focused on metabolic reprogramming in macrophages, their infiltration into metabolic organs, such as the liver, brain, pancreas, and adipose tissue, and their contribution to metabolic diseases [17]. In this sense, there is also a connection between diet and the role of adipose tissue as a tissue that is remodeled through changes in the expression of proinflammatory genes [18]. In obese adipose tissue, the epigenomic alterations [19] and macrophage-secreted products [20] contribute to the induction of tissue macrophages towards a proinflammatory (M1-polarized) phenotype involved in insulin resistance [21]. A new term, metaflammation, refers to the metabolic inflammation accompanying metabolic diseases; it is characterized by chronic inflammation driven by an excess of nutrients. The concept of ‘Metaflammation’ is now widely accepted [22].

In metabolic diseases, there are crossways between increased oxidative stress and chronic inflammation; the role of mitochondria is essential. Furthermore, some studies suggest that mitochondrial dysfunction and oxidative stress are involved in developing metabolic syndrome [23] and contribute to the progression of insulin resistance, inflammation, and cell death in various tissues and organs of the body [24]. These processes are particularly relevant in diabetes and metabolic diseases, where they accumulate glucose and fat in tissues such as the liver, muscle, and adipose tissue. Insulin resistance is a crucial feature of metabolic syndrome and T2DM, causing glucose to build up in the blood [25].

## 2. Oxidative Stress

There are a variety of sources of reactive oxygen species (ROS) in cells, including mitochondrial dysfunction and the activation of pro-oxidative enzymes (NADPH oxidases (NOX), xanthine oxidase (XO), lipoxygenase, myeloperoxidase (MP), and uncoupled oxide nitric synthase (eNOS)). At the same time, there are reductions in antioxidant systems. The mitochondria are a common site to produce ROS, due to the electron leak during the electron transport chain (ETC) function through the release of electrons from NADH and FADH2 at the complex I and III levels. Under conditions of hyperglycemia, the oxidative metabolism of the cell increases, and the accumulation of reducing equivalents (NADH+H, FADH2) feeds the respiratory chain with electrons, which leads to greater electron escape and increased superoxide radical (O_2_−•) production. The ROS can interact with other molecules or give rise to secondary ROS [26]. Superoxide can be converted to hydrogen peroxide (H_2_O_2_) by the enzyme Mn-dependent superoxide dismutase (SOD), which is present in the mitochondrial matrix. Excess H_2_O_2_ diffuses out of the mitochondria and is further metabolized by other antioxidant enzymes, such as cytosolic SOD, catalase, and glutathione peroxidase [27].

If antioxidant enzymes do not metabolize H_2_O_2_, they can interact with O_2^−^_• to produce the highly reactive and destructive hydroxyl radical (OH•) [28]. This radical can interact with nitric oxide (NO), producing a highly reactive peroxynitrite radical (ONOO) with reduced nitric oxide bioavailability in blood vessels, leading to vasoconstrictor effects. Recent research has identified monoamine oxidase (MAOs) enzymes in the mitochondrial outer membrane that can also generate H_2_O_2_ during hyperglycemic and proinflammatory states [29]. The increase in ROS in general, and O_2^−^_• in particular, generates activation of protein kinase C (PKC) and leads to activating oxidative stress signals (through NOX) and inflammation through the pathway nuclear transcription factor-kappa B (NF-kB). Studies in T2DM models demonstrate that aberrant NOX activation contributes to the uncoupling of eNOS, vasoconstriction, and endothelial dysfunction [30]. 

On the other side, decreased antioxidant activity is observed in metabolic diseases. Superoxide dismutase is a primary antioxidant defense in the cells, both in the mitochondria (Mn/SOD) and the cytosol (Cu/Zn/SOD). The decreased SOD activity was measured in animal models [30] and in most human studies [31]. In our group, we assessed cytoplasm SOD activity in patients with T2DM without and with vascular complications and detected that its activity was decreased in both groups of diabetics in comparison to the control group [32]. 

In conditions of hyperglycemia, up to 30% of glucose metabolism is derived from sorbitol formation by the enzyme aldose reductase, resulting in depletion of the cofactor nicotinamide adenine dinucleotide phosphate (NADPH) required for glutathione reductase (GRd) and eNOS functions. An increase in ROS production and a decrease in antioxidant systems (SOD, GRd) lead to oxidative stress and promote endothelial dysfunction (ED), which is a critical early step toward diabetic macrovascular complications [10,32]. In several studies, GPx activity was increased in diabetic patients with poor glycemic control compared with pre-diabetes, which can be considered as an adaptative response against free radicals [32,33]. Moreover, we reported that GPx activity exhibited high and significant risk value in developing vascular damage in diabetic patients over 40 years old and diagnosed with T2DM at least 5 years prior [32].

The latest studies suggest that lower activity of the Nrf2-Keap1 system (“nuclear factor-erythroid-2-related factor 2” and “kelch-like ECH-associated protein 1”) is also associated with metabolic disease’s development [34]. Nrf2 is a transcription factor that regulates the expression of antioxidant genes and plays a crucial role in protecting cells against oxidative stress [35]. It restricts oxidative stress by the feedback mechanism, causing superoxide detoxification, promotes the restoration of damaged DNA, and increases cell survival [36]. At the same time, Nrf2 regulates the intensity of the inflammatory process through the blockade of the NF-kB pathway and the production of proinflammatory cytokines [29]. Under oxidative stress conditions, Nrf2 can be activated and up-regulate the expression of various antioxidant enzymes, such as SOD, CAT, and GPx, to scavenge ROS and protect cells from oxidative damage.

Moreover, Nrf2 can protect vessels from injury by activating athero-protective genes, such as heme oxygenase-1 (HO-1), and Nrf2 has been reported to protect against ferroptosis, an iron- and ROS-dependent form of cell death [37]. The accumulation of advanced glycation end products (AGEs), through the activation of the receptor for AGEs (RAGE), inhibits Nrf2 signaling [38]. This interference results in reduced expression of Nrf2-targeted genes, leading to an impaired response to oxidative stress and increased susceptibility to diabetic and metabolic complications.

## 3. Mitochondrial Dysfunction in Diabetes and Metabolic Diseases

Mitochondrial dysfunction, conversely, refers to impaired mitochondrial function and energy metabolism, mitochondrial dynamic alterations, and pro-oxidative status [39]. Mitochondrial biogenesis is responsible for maintaining the number and size of mitochondria. This process is regulated by several transcriptional factors and enzymes and mediated via cellular stress or physiologic stimuli, such as temperature, physical exercise, dietary restriction, and muscle metabolism. In conditions of calorie restriction or exercise, silent information regulator 2 (SIRT1) and AMP-activated protein kinase (AMPK) regulate Peroxisome proliferator-activated receptor (PPAR)-γ coactivator-1α (PGC-1α) through deacetylation and phosphorylation [27]. Activation of PGC-1α leads to increased glucose utilization, mitochondrial biogenesis, fatty acid oxidation, and ROS neutralization [40,41]. On the other side, there must be a balance between the fission and fusion events of mitochondria [27]. In the condition of nutrient excess, this balance will be interrupted, and the dysfunctional mitochondria are not removed. As a result, an excessive generation of ROS, impaired calcium homeostasis, and decreased ATP production will occur [42]. Mitochondrial dynamic dysfunction plays a pivotal role in age-related diseases. Since metabolic diseases are adult diseases, this further aggravates mitochondrial dysfunction and oxidative stress [43,44]. It was demonstrated that mitochondrial biogenesis is reduced in the conditions of obesity and diabetes [45]. Disturbed mitochondrial dynamics increase the accumulations of mtDNA mutations, disturb antioxidant defense systems, and aggravate metabolic conditions, including metabolic diseases [46,47]. Increased ROS production leads to ROS-sensitive transient receptor potential melastatin 2 (TRPM2) channel activation in immune cells and Ca influx, and this mediates diabetic stress-induced mitochondrial fragmentation and compromises cell function, especially in the immune system [48]. Several studies reveal a close association between mitochondrial dysfunction and NLRP3 inflammasome activation [49]. In obesity, reducing the AMP-activated protein kinase (AMPK) AMPK reactivity leads to the inhibition of mitophagy and accumulation of damaged mitochondria and intracellular mtROS, release of mtDNA, and the NLRP3 inflammasome activation [50]. This, in turn, leads to IL-1β and IL-18 secretion and subsequent insulin resistance through serine phosphorylation of IRS-1 and impairment of insulin signaling [51,52]. Mitochondrial dysfunction can impair insulin signaling by altering ROS levels and ATP production. Moreover, ROS activates protein kinases such as JNK and IKK, which phosphorylate insulin receptor substrate-1 (IRS-1) and inhibit insulin signaling. Furthermore, reduced ATP production can impair insulin signaling by limiting the translocation of glucose transporters such as GLUT4 to the cell surface [53].

Regulation of mitochondrial enzymes and enzymes involved in fatty acid beta-oxidation, at the posttranscriptional level, plays an important role in regulating mitochondrial function. Mitochondrial dysfunction also leads to changes in the production and utilization of ATP and Acetyl-CoA, which can impact insulin signaling pathways. Notably, hyperacetylation of mitochondrial proteins reduces their activity, leading to mitochondrial dysfunction and lower ATP production [39]. For example, decreased ATP production can interfere with the activation of Akt, while altered acetyl-CoA levels can impact the acetylation of crucial proteins involved in insulin signaling [39]. This situation is produced in lipotoxicity conditions due to diet changes [54] or by the mitochondrial NAD-dependent protein deacetylase SIRT3 affected. Particularly, SIRT3 KO mice exhibited defective insulin-induced glucose uptake in skeletal muscle due to increased insulin resistance [55]. Alterations in mitochondria-organelle interactions often accompany mitochondrial dysfunction and insulin resistance. In particular, disruptions in mitochondria-lipid droplet communication have been shown to contribute to developing ectopic lipid accumulation, lipotoxicity, and insulin resistance in metabolic disorders such as obesity and T2DM [56]. 

Chronic low-grade inflammation has recently been proposed as a bridge between augmented fat accumulation and metabolic disorders, such as insulin resistance. Among several signaling involved pathways (NF-kB, p38 MAPK, JNK/SAPK), the activation of the nuclear factor-kappa B (NF-kB) transcription factor and the NLRP3 inflammasome (“the nucleotide-binding and oligomerization domain, leucine-rich repeat, and pyrin domain-containing 3”) play an essential role in metabolic diseases pathogenesis [57]. This pathway links oxidative stress, fat accumulation, and chronic low-grade inflammation with insulin resistance and the exacerbation of inflammation in metabolic diseases. Increased knowledge suggests that stressors such as high glucose levels and free fatty acids can activate the non-canonical NF-κB signaling pathway, producing cytokines and chemokines, promoting inflammation, and impairing β-cell function [58,59]. NF-kB is a primary factor in inflammatory reactions and diseases, connecting the metabolic, oxidative, immune, and inflammatory systems.

**Figure 1:**
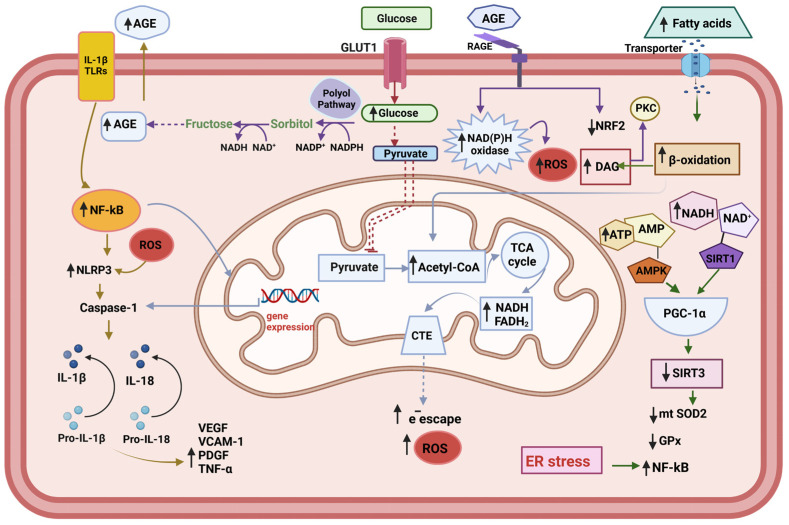
Metabolic changes in the endothelial cell. The endogenous increased cytokines act through the Toll-like receptors (TLRs) to activate NF-kB—NLRP3 inflammasome pathway. Under conditions of hyperglycemia, the accumulation of reducing equivalents (NADH+H, FADH2) feeds the respiratory chain with electrons, which leads to greater electron escape and increased superoxide radical (O_2^−^_•) production. The increment of polyol pathway leads to Advanced glycation end products (AGEs) accumulation, which act on the AGEs receptors (RAGE), leading to the activation of pro-oxidative enzymes, such as NAD(P)H oxidase, and decrement of NRF2, one important component of the anti-oxidative system of the cell. Increased β-oxidation upon the excess of fatty acid contributes to the accumulation of D-acyl glycerol, thus conducing to the activation of protein kinase C (PKC). The translocation of NF-κB to the nucleus activates the transcription of its target genes, including pro-IL-1, pro-il-18, and Pro-Caspase 1. The activation of NLRP3 inflammasome facilities the cleavage of the pro-Caspase 1 into its active form Caspase1, which in turn transforms IL-1β and IL-18 into their active forms. Subsequently, IL-18 induces the production of TNF-λ, which in turn promotes the synthesis and release of IL-6 and C reactive protein (CRP) (not shown). At the same time, the production of adhesion molecules is activated: vascular adhesion molecule-1 (VCAM-1), platelet-derived grown factor (PDGF), and vascular endothelial grown factor (VEGF). The endoplasmic reticulum (ER) stress contributes to the trigger for NLRP3 inflammasome activation and potentiating oxidative stress.

NF-κB is a transcriptional regulator that governs the transcription of genes implicated in immune system activity, inflammation, and cellular stress reactions [60]. Under normal conditions, NF-κB is sequestered in the cytoplasm by a protein complex called an inhibitor of kappa B (IκB) [61]. However, when cells are exposed to oxidative stress, reactive oxygen species can activate the IκB kinase (IKK) complex, which phosphorylates IκB, leading to its ubiquitination and degradation by the proteasome. This allows the translocation of NF-κB to the nucleus and activates the transcription of its target genes. One of the main downstream effects of NF-κB activation in metabolic diseases is the upregulation of proinflammatory cytokines, such as pro-IL-1β, and pro-IL-18. The activation of the NLRP3-ASC-pro-CASP1 complex facilitates pro-CASP1 cleavage for the active form of CASP1, which changes IL-1β and IL-18 into their active forms [62]. Subsequently, IL-18 induces the production of TNFα, which in turn promotes the synthesis and release of IL-6 and C reactive protein (CRP) [63]. One clear correlation exists between obesity, mitochondrial dysfunction, and activation of the NF-kB—NLRP3 pathway (Figure 1). Mice deficient in NLRP3 are protected from fat diet-induced obesity and insulin resistance [64]. 

High-fat diets produce changes in the intestinal microbiota and increase its permeability, leading to increased circulating levels of LPS and the proliferation of ceramides and saturated fatty acids. The NF-kB—NLRP3 inflammasome pathway can be activated by endogenous cytokines that increase during obesity and this activation is mediated by Toll-like receptors (TLRs). All these molecules act through TLRs. On the other hand, mitochondrial dysfunction increases the levels of oxidized mtDNA and ROS within the cytosol. These molecules may act as secondary signals for NLRP3 inflammasome activation [62,65]. IL-1β decreases IRS-1 tyrosine phosphorylation and its gene expression, inhibiting the insulin signaling pathway required for glycemic control and contributing to the development of insulin resistance [66]. The mechanism by which IL-6 and TNF-α contribute to insulin resistance is complex and involves several pathways. One proposed mechanism is that these cytokines activate the serine/threonine kinase phosphatidylinositol 3-kinase/protein kinase B (PI3K/AKT) and can activate the Janus kinase/signal transducer and activator of the transcription 3 (JAK/STAT) pathway. In both cases, insulin receptor substrate-1 (IRS-1) phosphorylation inhibits insulin signaling and promotes insulin resistance [67,68]. An animal model study demonstrated that retinal vascular leakage was significantly reduced in diabetes-induced mice, as well as in vitro, on cultured cells, using a specific inhibitor of the JAK/STAT pathway; the study’s authors suggested that inhibiting this pathway could be a promising approach to the treatment of diabetic retinopathy [69]. Additionally, IL-6 and TNF-α can induce the production of other cytokines, such as monocyte chemoattractant protein-1 (MCP-1) and fractalkine [70]. 

In another approach, TLRs activation initiates a cascade of signaling events that lead to the expression of inflammatory cytokines, chemokines, and other effectors molecules, ultimately leading to the activation and recruitment of immune cells to fight off invading pathogens or promote tissue repair [71]. For example, TLR4 has been shown to impair insulin signaling and glucose uptake in adipocytes, while TLR2 and TLR9 have been shown to modulate insulin secretion in β-cells [72]. The TLR4 is crucial in initiating proinflammatory reactions. TLR4 activation can lead to the recruitment of MyD88, leading to NF-κB pathway activation [71]. 

Evidence indicates that endoplasmic reticulum (ER) stress is a crucial trigger of NLRP3 inflammasome activation and is potentiated by oxidative stress, which is a cellular response to the accumulation of unfolded or misfolded proteins in the ER [73]. However, if the ER`s stress is too severe or prolonged, the UPR can also activate pro-apoptotic pathways and lead to β-cell death [74]. These interconnected processes can lead to a vicious cycle of oxidative and ER stress, promoting the development of cardiovascular diseases.

As a result, the production of IL-1β, IL-18, IL-6, and TNF-α increases [75]. These cytokines are released into the extracellular space and act as paracrine molecules on endothelial cells (ECs), promoting the expression of endothelial adhesion molecules such as intercellular adhesion molecule-1 (ICAM-1), vascular adhesion molecule-1 (VCAM-1), increasing the procoagulant activity. IL-1β decreases IRS-1tyrosine phosphorylation and its gene expression, contributing to the development of insulin resistance [66]. Recently, overexpression of proinflammatory genes (IL-1β, IL-6, TNF-α, and VEGF) and profibrotic genes (ICAM-1, VCAM-1) has been correlated with the appearance of nephropathies in diabetic patients [76]. 

## 4. Regulation of Oxidative Stress and Inflammation by microRNAs

MicroRNAs (miRNAs) are small non-coding molecules containing approximately 18–22 nucleotides and can regulate the expression of their target mRNAs at the posttranscriptional level, and their expression can be modified by external agents and medication [77]. This review will focus on miRNAs involved in regulating oxidative stress and the NF-kB—NLRP3 inflammasome pathway. 

Research on miRNAs in recent years has drawn attention to their critical function in disease diagnosis and intercellular communication. Extracellular miRNAs have emerged as key players in mediating communication between different tissues and have the potential to serve as valuable markers for diagnosing various diseases. The amount of miRNA in each cell is determined by the combination of endogenous miRNA production within the cell and the uptake of exogenous miRNAs from the extracellular environment [78,79]. Both factors contribute to the total miRNA content in a cell and can impact cellular functions and gene expression.

Accumulating evidence suggests that extracellular vesicles (EVs), mainly exosomes, can propagate inflammation and participate in the development of vascular damage [80]. The effect of EVs released by inflamed endothelial cells (ECs) on the reprogramming of monocytes toward a proinflammatory phenotype has recently been studied [81]. In vitro studies on ECs exposed to hyperglycemic (HG) conditions demonstrated that the number and size of EVs change and that they have greater procoagulant activity, contributing to progressive endothelial injury [82]. Interestingly, the senescence similarly affected EVs’ properties and miRNAs’ cargo [83]. Zhang et al. observed that adipocyte-derived EVs from obese mice significantly enhanced macrophage proinflammatory phenotype in adipocytes, probably due to the upregulation of miR-155 [84]. Castaño et al. showed that EVs isolated from the plasma of obese mice induced glucose intolerance and dyslipidemia in lean mice. These dysfunctions may be caused by increased levels of exosomal miR-122, miR-192, and miR-27 [85].

Several studies have revealed that oxidative stress can alter the expression levels of many miRNAs [86,87], suggesting that miRNAs may be involved in the cellular response to oxidative stress. Additionally, some miRNAs have been identified as regulators of oxidative stress in the cardiovascular system by targeting ROS generators, antioxidant signaling pathways, and selected antioxidant effectors [88]. For example, miR-21 has been shown to target several genes involved in oxidative stress, including superoxide dismutase (SOD) and heme oxygenase-1 (HO-1). miR-126 induces SIRT1 and SOD2 expression, protecting endothelial cells against ROS production and senescence [89]. MiR-140-5p decreased oxidative stress and ROS levels by increasing the protein expression of nuclear factor-erythroid 2-related factor 2 (NRF2) and sirtuin-2 (SIRT-2), and HO-1 [90].

As we have described, inflammation plays a vital role in developing metabolic diseases. In recent years, miRNAs have emerged as key regulators of inflammation, playing critical roles in modulating the signaling pathways that control the onset and termination of inflammation [91]. Several miRNAs have been identified as essential players in the pathogenesis of inflammatory diseases, and manipulating their expression levels holds promise as a clinically applicable therapy. Among the most studied miRNAs are miR-21, miR-146a, miR-10a, miR-9, and miR-155, which participate in the epigenetic regulation of mitochondrial dysfunction and inflammation at different levels. miR-21 and miR-9 have an inflammatory effect that may result from their ability to promote activation of the NF-kB and NLRP3 [92,93]. miR-146a acts directly on energy metabolism at the mitochondrial level, and its low expression is related to chronic inflammation levels in macrophages [94]. MiR-155 is involved in the modulation of inflammation, including in obesity, atherosclerosis, and diabetes [95]. miR-126 is related to regulating the insulin receptor in adipose tissue and maintaining vascular integrity and angiogenesis [96]. In one recent study, circulating miR-21, miR-126, and GPx and AOPP levels showed an acceptable predictive value and were considered possible biomarkers of vascular damage in T2DM patients, but more studies are needed [32].

Recent studies have revealed that obesity can impact the content of EVs, including exosomes, which are secreted by adipose tissue and can carry miRNAs as part of their cargo. For example, EVs derived from adipose tissue taken from obese and lean individuals have been shown to contain different miRNA profiles [97,98]. Moreover, miRNA content in EVs has been shown to differ between African American females pre-and post-bariatric surgery [99], suggesting that obesity and its treatment can influence miRNA packaging into EVs. Studies comparing obese and lean mice have also shown changes in EVs and miRNAs [100]. Notably, adipose tissue macrophage-derived exosomes, termed ATM-EXOs, isolated from obese mice have been shown to confer insulin resistance and glucose intolerance when injected into lean mice. This suggests that EVs derived from adipose tissue in obesity can influence metabolic homeostasis in recipient cells or tissues.

Besides, improvement in insulin resistance was observed when ATM-EXOs from lean mice were injected into obese mice [100]. The study showed that miR-155 was enriched in ATM-EXOs from obese mice compared to lean mice, and its inhibition in ATM-EXOs attenuated the detrimental effects on glucose homeostasis. Furthermore, insulin resistance was decreased when ATM-EXOs from lean mice, which had lower miR-155 levels, were injected into obese mice [100]. This suggests that miR-155 in EVs may contribute to the metabolic effects of obesity, and its modulation could potentially be a therapeutic strategy for metabolic disorders associated with obesity. The adipose tissue macrophages secreted EVs with increased content of miR-155 [20]. Knockout of miR-155 in obese mice improved insulin sensitivity and glucose tolerance compared to controls, suggesting that miR-155 can be transported to insulin target cells and have a robust effect on insulin sensitivity [20]. However, the role of miR-155 in regulating the NLRP3 inflammasome is still unknown. A recent study using in vitro and the atherosclerosis models (ApoE−/− mice) demonstrated that miR-155 participates in NLRP3 inflammasome activation in ox-LDL-induced macrophages via the ERK1/2 pathway [101].

**Figure 2:**
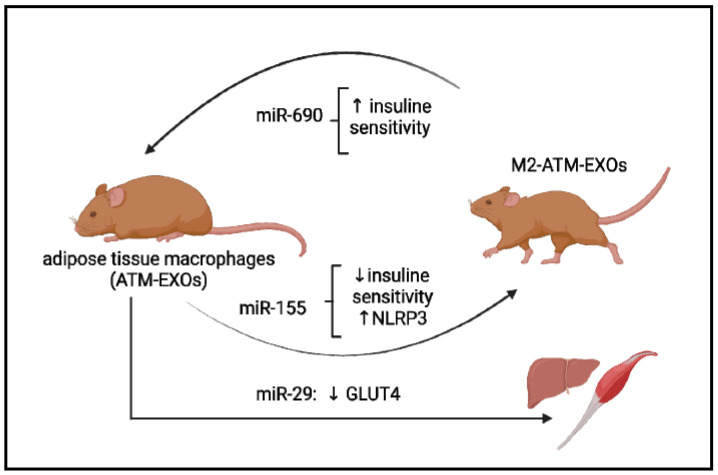
Some examples of the exosome’s microRNAs’ participation in the pathogenesis of metabolic diseases. Exosomes derived from adipose tissue macrophages, designated ATM-EXO, isolated from obese mice have been shown to confer insulin resistance and glucose intolerance when injected into lean mice (achieved in miR-155). ATM-derived miR-29 can transfer to myocytes, hepatocytes, and adipocytes, causing insulin resistance in vivo. miR-690, abundant in exosomes of anti-inflammatory M2-like macrophages, repolarized ATM-EXO towards an anti-inflammatory M2-like phenotype.

On the contrary, miR-690 was the most abundant miRNA expressed in exosomes from anti-inflammatory M2-like bone marrow-derived macrophages (M2 Exos) [102]. The authors of this study assessed the effect of Exos on the in vitro and in vivo models and concluded that treatment with M2 Exos repolarized the activation state of ATMs toward an anti-inflammatory M2-like phenotype (Figure 2). Moreover, using miR-690 mimic agents, the authors demonstrated that miR-690-enriched EXOs could promote insulin sensitivity in obese mice (Figure 2) [102]. In turn, other miRNAs are involved in metabolic memory maintenance at the epigenetic level.

MiR-29a is another candidate for regulating obesity-associated insulin resistance [103]. MiR-29a is increased in exosomes derived from adipose tissue macrophages and can be transferred into myocytes, hepatocytes, and adipocytes, causing insulin resistance in vitro and in vivo (Figure 2) [103]. The miR-29 family contains three miRNAs: miR-29a, miR-29b, and miR-29c. It has been shown that miR-29a and miR-29c regulate GLUT4 receptor expression in skeletal muscle. The overexpression of these miRNAs decreases glucose entry under basal conditions, and when insulin is stimulated, the metabolic processes that take place during glycolysis and gluconeogenesis are reduced [104]. Hyperglycemia induced metabolic memory in endothelial cells of the diabetic animal model, leading to upregulation of miR-27a-3p, down-regulation of NRF2 expression, increased transforming growth factor-β (TGF-β) signaling, as well as generating ROS. All these changes are involved in cardiac dysfunction in diabetes and are not erased when switched to a low glucose level [105]. According to this in vitro study, miR-27-3p is a key regulator for the down-regulation of NRF2 induced by hyperglycemia, and it is involved in metabolic memory. It also was demonstrated that treatment by the NRF2 activator and miR-27a-3p inhibitor has improved myocardium function in a diabetic mouse model [105]. So, a novel signaling pathway involving an NF-kB/miR-27a-3p/NRF2/ROS was proposed as a novel target for blocking metabolic memory.

MiR-10a inhibits multiple target genes involved in the NF-κB signaling pathway. Down-regulation of miR-10a has been observed in inflammatory conditions, suggesting that targeting inflammatory responses through miR-10a mimic could be an effective therapeutic strategy [91]. In animal model studies, miR-10a mimic has been shown to attenuate inflammation in various disease models, including diabetic kidney disease [106]. Further research is needed to explore the therapeutic potential of miR-10a mimic in clinical settings, including metabolic diseases. MiR-9 is another miRNA that has been implicated in the regulation of inflammation. It has been shown to be up-regulated in human monocytes and neutrophils upon NF-κB activation and acts as feedback control of the NF-κB-dependent responses. MiR-9 has been shown to inhibit the formation of the inflammasome NLRP3 [107], likely via targeting JAK1/STAT1 signaling [107]. Further studies are warranted to elucidate the precise mechanisms underlying the regulatory effects of miR-9 in inflammation and explore its therapeutic potential.

MiR-146a-5p decreased expression was detected in HAECs in hyperglycemic conditions. Furthermore, its decrease was persistent even when these cells were not in the conditions of the previous high glucose [108]. miR-146a targets TRAF6 and IRAK1, inhibiting NF-kB binding activity [109], and is involved in the expression of pro-atherogenic genes, namely, MCR-1 and IL-6 [27]. A significant age-related decline in c-miR-146a was reported in healthy subjects and T2DM patients [110]. Moreover, this miRNA was proposed as a novel age-related biomarker of healthy/unhealthy aging trajectories [94].

The circulating miRNA signature and exosome-containing miRNAs are strongly modulated during the natural history of the disease and could be affected by treatments applied to patients. Moreover, new data suggest that using different therapies or supplements that modulate the miRNA load, together with the standard treatment, can be an effective way to improve the patient’s condition and prevent the appearance of vascular complications.

## 5. Impact of Treatments on Oxidative Stress and microRNA Expression in Metabolic Diseases

Metformin is the first-line drug in managing type 2 diabetes mellitus (T2DM) due to its easy availability, with more than 60 years in the market [111], and its pleiotropic characteristics on the organism make it a drug used globally. Previous studies demonstrated that metformin treatment influences the biology of the endothelium (angiogenesis, senescence, proliferation, migration, and capillary tube formation) [112,113,114] and influences several miRNA expressions [110]. One of the actions of metformin is the upregulation of miR-146a [95], miR-99b [113], and down-regulation of miR-155 [113]. Mensa et al. also demonstrated that miR-146a was significantly overexpressed in T2DM patients treated with metformin [94]. Considering that this miRNA is involved in the down-regulation of the NF-kB pathway, this effect favors the emerging anti-inflammatory and anti-aging pleiotropic effect of metformin [115]. Moreover, miR-99b and miR-155 regulate cardiac hypertrophy via the upregulation of AKT, pointing to the anti-hypertrophic role of metformin. Cytoprotective effect of metformin was demonstrated by the activation of the nuclear factor-erythroid 2-related factor (Nrf2)/heme oxygenase (HO)-1 pathway, which is dependent on AKT activation by this drug [116]. Three months of metformin therapy for type 2 diabetes mellitus patients demonstrated effective inhibition against glycation and receptor RAGE-mediated cellular inflammation and increased antioxidant levels [117]. More studies are needed to confirm the mechanism of metformin in metabolic disease treatment, especially its role in restoring mitochondrial dysfunction and reducing oxidative stress and cytokine production.

Empagliflozin is a drug with an antihyperglycemic effect, and it is the inhibitor of the sodium-glucose cotransporter 2 (SGLT2), indicated for patients with T2DM and chronic symptomatic heart failure [118]. It is well described in several studies in vitro, protecting the role of this drug on endothelial function. In patients, a recent study conducted by Mone P. et al. evaluated the expression of a complete set of microRNAs related to endothelial function; two groups of patients were recruited, the first one with T2DM, frailty, and preserved ejection fraction (HFpEF). They were treated for 3 months with empagliflozin, metformin, or insulin [119]. The second one was a healthy age-matched control; their results indicated a novel microRNA signature for five miRNAs significantly regulated in HFpEF patients versus healthy control subjects (two up-regulated corresponding to miR-21 and miR-92 and three down-regulated corresponding to miR-126, miR-342-3p, and miR-638). This finding could ameliorate endothelial function and modify the course of the macrovascular complication of T2DM.

Another group of therapeutic drugs called Dipeptidyl peptidase 4 (DPP-4) inhibitors/gliptins have been used since 2006 when Food and Drug Administration (FDA) and European Medicines Agency (EMA) agencies approved their use for T2DM as the second or third-line medication after metformin with sulfonylureas (SU). The action mechanism of these drugs is blocking the catabolism of incretin hormones, including GLP-1 (glucagon-like peptide-1) secreted in distal small intestinal by neuro-endocrine L cells and GIP (gastric inhibitory peptide) secreted in the stomach and proximal small intestine by the neuro-endocrine K cell. Until recently, few studies have explored miRNAs’ role in humans, researchers have mainly developed in vitro and animal models for diabetes mellitus [120]; the data available to humans are provided by Catanzaro et al. They recruited 40 elderly patients (>65 years) with metformin as the basal medication and hemoglobin A1c (HbA1c) levels of 7.5–9.0%. Under this criterion, they added sitagliptin (100 mg once daily). After 3 and 15 months of following up, they divided patients into no-responders (NR) and responders (R), corresponding to 15% and 85% of the population, respectively. The variable considered for this classification was good glycemic control with a <7.5 HbA1c level cutoff point or less than 0.5% at the basal sample. Their results are addressed to two miRNAs with positive responses to sitagliptin: miR-126-3p and miR-223. The latter is important for regulatory T cells, neutrophils, and monocytes since possibly increased circulation improves the immune system, cell survival, and wound healing in T2DM patients. On the other hand, miR-338 was proposed for these authors to be a candidate for measuring resistance to gliptins. In another study performed by Younis et al., 60 T2DM patients with coronary artery disease (CAD) were classified in a ratio of 2:1, 40 patients received vildagliptin/metformin, and the other 20 patients only received metformin. IL-1β, HbA1c, and high sensitivity C reactive protein (hsCRP) were measured under baseline conditions and repeated 12 weeks after treatment. Mainly results indicate that IL-1 β, hsCRP, and HbA1c were significantly lower in the vildagliptin/metformin group, and inverse manner increased levels of the same biomarkers were observed in the metformin group. FDA recognizes gliptins as safe cardiovascular drugs [121] and maybe one possible route is the up-regulated mir-223 for decreased cytokines proinflammatories such as IL-1 β. 

Finally, thiazolidinediones (TZDs), Rosiglitazone, and Pioglitazone are PPARs (peroxisome proliferator-activated receptor gammas), whose mechanism of action increases insulin sensitivity and is unique to these properties. PPAR expression is high in adipose tissue, and in other tissues, it alters miRNA-containing extracellular vesicles derived from cancer. The PPAR variant, PPARγ, is most important in lipogenesis and lipid biosynthesis, with the highest expression in white adipose tissue. These drugs emerged from 1999 until today and are widely used as the second line in treating T2DM. Some studies developed in humans reveal an association between some miRNAs and the response to TZD. In 2015, Flowers et al. divided ninety-three non-diabetes patients into insulin-resistant and non-insulin-resistant groups. Their criteria for insulin resistance and insulin sensitivity were determined as a steady-state plasma glucose (SSPG) level of ≥180 mg/dL and <120 mg/dL, respectively. At the baseline of 75 individuals that were insulin-resistant, they found that 5 miRNAs were differentially expressed: miR-193b (1.45 fold change (FC)), miR-22-3p (1.15 FC), miR-320a (1.36 FC), miR-375 (0.59 FC), and miR-486 (1.21 FC) (all *p* < 0.05) respecting to the sensitive group (n = 18). On the other hand, all participants received different dosages of rosiglitazone and pioglitazone at different times [122]. Fifty-five participants were medicated with 4 mg rosiglitazone for 4 weeks and 8 mg rosiglitazone for 8 weeks, and thirty-eight participants received 15 mg pioglitazone for 2 weeks, respectively, then 30 mg pioglitazone for 2 weeks, followed by 45 mg for 8 weeks. In the end, 36 participants were insulin-resistant, denominated responders showed improved insulin sensitivity, and 6 miRNAs were differentially expressed between responders compared to non-responders (11 participants) (miR-20b-5p (1.20 FC), miR-21-5p, (0.92 FC), miR-214-3p (1.13 FC), miR-22-3p (1.14 FC), miR-320a (0.98 FC), and miR-486-5p (1.25 FC) (all *p* < 0.05)). Nevertheless, only miR-320a and miR-486-5p identified the insulin-resistant group’s responders to thiazolidinedione administration, and those miRNAs are closely associated with insulin resistance [122]. 

The most recent work by Nunez et al. proposed that pioglitazone treatment influences the content of extracellular vesicles (EVs) [123]. In this work, miRNAs were isolated from circulating plasma exosomes and the adipose tissue in 24 T2DM patients treated with pioglitazone 45mg and a placebo for three months. The expression levels of 5 miRNA in circulating exosomes (EV-miR-374b-5p, EV-miR-195-5p, EV-miR-20a-5p, and EVmiR-7-5p, and EV-miR-92a-3p) were decreased after the administration of pioglitazone. However, miR-195-5p was overexpressed by pioglitazone in adipose tissue, one of the main targets of the drug. A correlation was also observed between EV-miR-374b-5p and EV-miR-195-5p, suggesting the coregulation of both microRNAs. Among the genes over-directed by miRNA, RAF1 decreases its expression in adipose tissue by applying pioglitazone. This enzyme is a positive regulator of lipolysis in adipocytes [124]; thus, the increase in miR-195-5p in adipose tissue may induce the suppression of lipolysis, leading to weight gain but improving insulin sensitivity.

Melatonin (N-acetyl-5-methoxytryptamine) can enter the mitochondria, which increases electron transport chain activity and ATP production, conducing to the regulating mitochondrial membrane potential and preventing mPTP opening. It limits the escape of cytochrome c and the activation of apoptosis. Mitochondria-produced ROS are directly neutralized by melatonin and also by its metabolites N1-acetyl-N2-formyl-5-methoxykynuramine (AFMK) and N-acetyl-5-methoxykynuramine (AMK) [125]. Moreover, melatonin at the genetic level activates the expression of antioxidant enzymes and reduces the expression of iNOS and other proinflammatory molecules. The decrease in oxidative stress contributes to reducing inflammation, adds to the ability of melatonin to inhibit the NF-κB pathway and NLRP3 [126,127], and restores the antioxidant capacity of Nrf2 during aging [128]. Recent studies, including ours, show that melatonin influences the expression of miRNAs, including inflamma- and mito-miRNAs [129]. In studied animal diabetic models, melatonin acts as a heart-protector [130] and as an anti-apoptotic protector in the brain [131]; it also decreases renal inflammation and fibrosis, inhibiting the NF-kB and TGF-β1/Smad3 pathways, both in an animal model and in the culture of mesenchymal cells [132]. Under HG conditions in HUVEC, melatonin has an anti-apoptotic effect through the PI3K/Akt, Bcl-2, and oxLDL/LOX-1 pathway, as it influences endothelial progenitor cells (EPC) from healthy human donors cultured under HG conditions (25 mM). In this case, treatment with melatonin increased eNOS phosphorylation and HO-1 expression [133]. More studies are needed to evaluate the impact of melatonin on miRNAs’ cargo and EVs.

In the following table (Table 1), we resume some of the treatments directed at oxidative and inflammatory markers and microRNA expression:

## 6. Conclusions

It has been several years since miRNAs began to be studied by different groups of researchers worldwide. miRNAs have been evaluated in various in vitro and in vivo research models and in T2DM patients with various pharmacological treatments and surgical approaches. However, until recently, miRNAs were not considered a predictor biomarker for DM progressions, like HbAc1, HOMA-IR, fasting glucose, and BMI. More studies are also needed in this direction. Perhaps a possible strategy to achieve its recognition as a promising predictor and therapeutical biomarker is to disseminate information about miRNAs highly associated with macrovascular complications in clinical settings, thus cultivating a genuine interest in all those family clinic physicians, who initially receive and treat patients with diabetes mellitus. Since they generally focus on giving lifestyle recommendations and proper nutrition, they lose their motivation to study their patients and the biochemical markers described for more than 20 years. On the other hand, it is also essential to sensitize international health organizations to the use of these novel biomarkers to control the high incidence rate of diabetes mellitus since, according to the OMS, is not decreasing but rather increasing along with its complications (hemodialysis, coronary angioplasties, amputations of pelvic limbs, etc.). These therapeutic interventions are more expensive than finding a more appropriate pharmacotherapy for each patient and treating these patients’ micro- and macrovascular complications.

## Figures and Tables

**Table 1:** The impact of different metabolic disease interventions on oxidative and inflammatory markers and miRNAs.

Supplement or Treatment/ Type of Metabolic State and Model Study	Impact on Different Markers and miRNAs Expression	Authors of Study
Resveratrol supplementation with 200 mg for 24 weeks in diabetic patients (n = 110). It was a randomized, double-blinded, placebo-controlled parallel-group trial.	A significant anti-inflammatory effect by demonstrating a marked reduction in IL-6 and TNF-α and improving glycemic control by reducing insulin resistance. Changes in miR-34a, miR-375, miR-21, and miR-192 (down-regulation) and changes in miR-126 and miR-132 (upregulation).	[134]
Ginkgolide B (GB), the active ingredient of Ginkgo biloba, was used in doses of 200 mg/kg/d in diabetic-induced mice for 12 weeks. Mouse renal podocytes (MPC5) in vitro model were treated in modeling concentrations of 1.5 mM palmitic acid + 25 mM glucose. Effects of 80 μM GB on ferroptosis markers were studied.	In animal models, GB treatment reduced serum total cholesterol and reduced hyperglycemia. GB may inhibit oxidative stress and ferroptosis by inhibiting GPX4 ubiquitination, preventing diabetic nephropathy progression. In a culture of mouse renal podocytes under hyperglycemic and hyperlipidemic conditions, GB inhibited the expression of ferroptosis markers TfR1 and fibrosis markers α-SMA and Collagen α1.	[135]
Effect of aerobic training (AT) and vitamin D supplementation in patients with T2DM on oxidative stress and inflammatory markers. Notably, 48 men were involved in a single-blinded, randomized, placebo-controlled trial for 8 weeks. AT for 20 min at a 60% heart rate maximum per session as they were progressing to 40 min. Notably, 50,000 IU of Vit D supplements capsules per week.	The oxidative stress and glucose markers (Fasting Blood Glucose (FBG) and Homeostasis Model Assessment of Insulin Resistance (HOMA-IR)) were improved in plasma. Moreover, it was assayed, which significantly down-regulated the gene expression of TNF-α, IL-1β, MAPK1, and NF-kB in Peripheral Blood Mononuclear Cells (PBMCs).	[136]
Animal models in rats injected with streptozotocin (diabetic model) were divided into the model group and miR-375 inhibitor group versus the control group (n = 10 per each group). The experiment lasted 3 months; mir-375 siRNA was injected into the tail vein every 3 days. Rats developed diabetic retinopathy (DR).	MiR-375 siRNA decreased the serum malondialdehyde (MDA) content and increased the SOD activity in DR rats. MiR-375 inhibitors reduced blood glucose, retinal permeability, and optic ganglion apoptosis in rats with DR. MiR-375 siRNA repressed Ndrg2, IL-6, and STAT3 mRNA protein expression levels in the retinal tissue of DR rats. The mechanism of action may be related to the regulation of the Ndrg2/IL-6/STAT3 signaling pathway.	[137]

## Data Availability

Not applicable.
